# High Performance Mg Alloy with Designed Microstructure and Phases

**DOI:** 10.3390/ma17112734

**Published:** 2024-06-04

**Authors:** Zhao Yang, Chao Xu, Shengnan Song, Taiki Nakata, Shigeharu Kamado

**Affiliations:** 1 School of Materials Science and Engineering, Harbin Institute of Technology, Harbin 150001, China; zhaoyang@ahpu.edu.cn (Z.Y.); selene95@163.com (S.S.); 2 School of Materials Science and Engineering, Anhui Polytechnic University, Wuhu 241000, China; 3 Research Center for Advanced Magnesium Technology, Nagaoka University of Technology, Nagaoka 940-2188, Japan; nakata@mech.nagaokaut.ac.jp (T.N.); kamado@mech.nagaokaut.ac.jp (S.K.)

**Keywords:** magnesium alloys, microstructures, mechanical properties, local strain evolution, crack propagation

## Abstract

A high strength and ductile Mg-Gd-Y-Zn-Zr alloy was designed and fabricated. The local strain evolution of the alloys during plastic deformation was analyzed using high-resolution digital image correlation (DIC). The results showed that the β particles, nano-sized γ’ phases, and LPSO phases were distributed in the as-extruded alloy and a bimodal microstructure was exhibited, including elongated un-dynamic recrystallized grains and fine dynamic recrystallized grains. With increasing extrusion ratio, the grain size remained, with the volume fraction of dynamic recrystallization of the as-extruded alloy increasing from 30% to 75%, and the as-extruded alloy exhibited a high strength-ductility synergy, which is attributed to the grain refinement, extensive β particles, and elongated block-shaped LPSO phases. The strain evolution analysis showed that a strain-transfer from un-DRXed regions to adjacent DRXed regions and LPSO phases can promote uniform plastic deformation, which tends to improve the ductility of the alloy.

## 1. Introduction

With growing concerns for light weighting and power saving in the automobile and aerospace industries, magnesium (Mg) alloys have attracted extensive attention due to their obvious advantages of low density and high specific strength [1,2,3]. However, the conventional drawbacks of Mg alloys severely restrict the application of Mg alloys, such as their low strength and poor ductility [4]. Recently, the addition of rare earth (RE) elements into Mg alloys has been regarded as a reliable method to improve the mechanical performance of Mg alloys [5,6]. As such, the Mg-RE-Mn alloy and Mg-RE-Zr alloy fabricated by Rokhlin et al. [7] and Kamado et al. [8] show high specific strength and excellent creep resistance compared to conventional Mg alloys [9]. Yu et al. investigated the high-strength Mg-Gd-Y-Nd-Zr alloy, which exhibited an excellent tensile yield strength (TYS) of 500 MPa, strengthened via a bimodal microstructure and nano-scale β’ precipitates [10]. Moreover, the addition of moderate amounts of Zn to the Mg-Gd-Y alloy, forming long-period stacking ordered (LPSO) phases, could improve the balance between strength and ductility [11]. For example, the Mg-Gd-Y-Zn-Zr alloy has a high ultimate tensile strength of 493 MPa, owing to the pinning effect of dislocation strengthening and precipitation strengthening [12]. Wang et al. reported that the Mg-RE-Zn-Mn alloy with lamellar LPSO and nano β’ phases achieved superior mechanical properties with an ultra-high yield strength of 420 MPa and a moderate ductility of 6.3% [13].

Therefore, the bimodal microstructure and LPSO phases play an active part in strength–ductility balance, and the effect of a bimodal microstructure and LPSO phases on the mechanical properties of the Mg-RE alloy containing LPSO phases needs to be further investigated.

Several recent research studies have been devoted to the issue on the effect of the LPSO phases on high performance in the Mg-RE alloy. It was found that, during thermomechanical processing, the block 18R LPSO phases could be elongated along the extrusion direction, and the lamellar 14H LPSO phases kink obviously and promote recrystallization behavior in the Mg-Y-Zn-Li alloy, thus improving both its strength and ductility [14]. Meanwhile, the basal dislocation pile-up easily lies at the interface of the α-Mg matrix and the LPSO phases, thus decreasing deformation twins, which is conducive to enhancing ductility [15]. However, few studies have focused on inhomogeneous strain distribution around the LPSO phases in Mg-RE alloys during plastic deformation. The study of the local strain distribution in Mg-RE alloys with a multi microstructure and second phases will contribute to improving their mechanical properties.

In this study, Mg-Gd-Y-Zn-Zr alloys containing a multi microstructure and second phases were prepared. The microstructure evolution of the alloys and its effect on mechanical properties were systematically investigated. The strain evolution of as-extruded alloys during plastic deformation were observed by means of digital image correlation (DIC).

## 2. Materials and Methods

A water-cooled cast Mg-8Gd-4Y-1Zn-0.4Zr (wt.%) alloy ingot was used. Billets with a diameter of 43 mm and a height of 38 mm were cut from as-cast ingots. The billets were homogenized at 510 °C for 12 h, followed by water quenching (~60 °C). The homogenized billets were held in 400 °C for 5 min, and then extruded with a ram speed of 0.1 mm s^−1^. The extrusion ratios were 5, 7.5, and 10, respectively, which were denoted as R5, R7.5, and R10. Finally, the as-extruded rods were aged at 200 °C for 72 h, followed by water quenching.

Microstructures of samples were observed using an optical microscope (OM, BX53M, Olympus, Tokyo, Japan) and a scanning electron microscope (SEM, Merlin Compac, ZEISS, Jena, Germany) in combination with electron back-scattered diffraction (EBSD), and the corresponding EBSD data were analyzed with OIM software (OIM 6.5, VIC-2D 2009). The EBSD-analyzed area and step size were 150 × 150 μm^2^ and 0.3 μm, respectively. An X-ray diffractometer (XRD, Panalytical, Empyrean, Amsterdam, The Netherlands) was used to identify phases in the alloys. For the tensile test, a Shimadzu Autograph AG-X Plus machine (Shimadzu, Kyoto, Japan) was employed at room temperature with an initial strain rate of 10^−3^ s^−1^. Specimens for the tensile test were 15 mm in length and 4.5 mm in width, which is referenced in the Chinese Standard GB/T 228.1-2010 [16]. To monitor local strain distribution, the EBSD and DIC methods with VIC-2D software were used during the tensile test.

## 3. Results

### 3.1. Microstructures before Hot Extrusion

**Figure 1:**
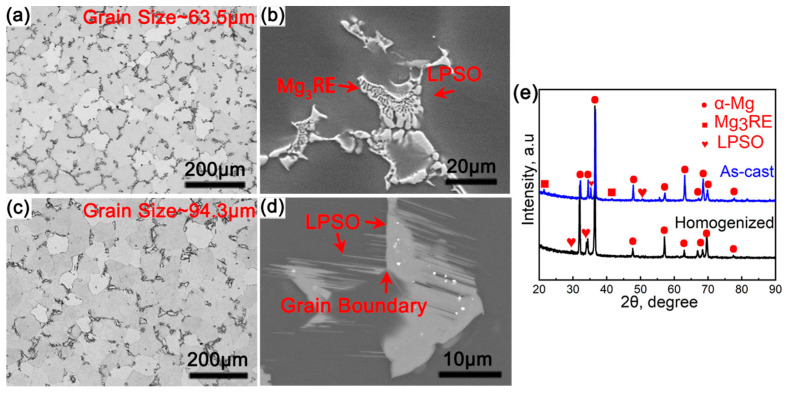
Microstructures of the (**a**,**b**) as-cast and (**c**,**d**) homogenized alloys: (**a**,**c**) OM images and (**b**,**d**) SEM images; (**e**) shows the X-ray diffraction patterns of the studied alloys.

Figure 1a,b show the microstructure of the as-cast Mg-8Gd-4Y-1Zn-0.4Zr sample. The grain size of the as-cast sample was ~63.5 μm, the discontinuous eutectic phases were distributed along grain boundaries, and the lamellar-shaped phases formed around the eutectic phases. After homogenization, the grain size was slightly increased (~94.3 μm), and the morphology of grain boundary phases was changed into dendritic intergranular block-shaped and intragranular thin plate-shaped (Figure 1c,d). Combined with previous research and the corresponding XRD results (Figure 1e) [17,18], these second phases in the as-cast and homogenized samples were Mg3RE phases (FCC, a = 0.732 nm) [19] and LPSO phases, respectively. The block-shaped LPSO phases were transformed from non-equilibrium Mg3RE phases due to the diffusion of solute atoms [20]. The precipitation of the lamellar-shaped LPSO phases in the homogenized sample was accompanied by the dissolution of the eutectic phases [21].

### 3.2. Microstructures of the As-Extruded Samples

**Figure 2:**
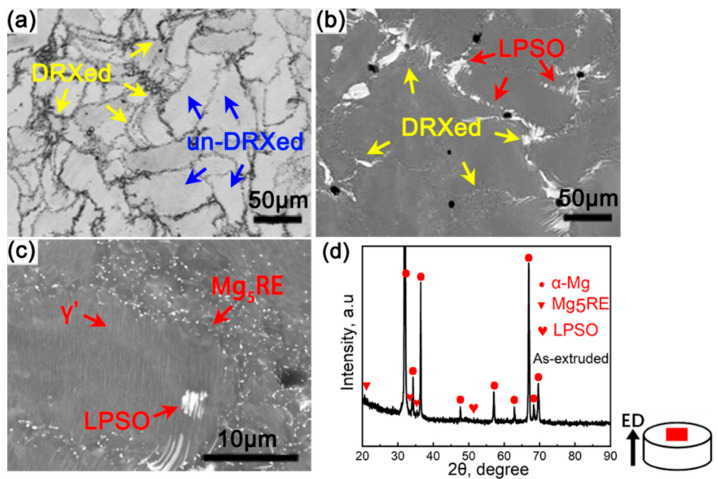
Microstructures of the as-extruded alloys, using the extrusion ratio of 7.5: (**a**) OM image and (**b**,**c**) SEM images; (**d**) shows the X-ray diffraction patterns of the as-extruded alloys.

Figure 2a–c show the bimodal microstructure of the as-extruded sample, using the extrusion ratio of 7.5, consisting of fine DRXed grains and coarse un-DRXed grains. After hot extrusion, the undissolved LPSO phases were broken and remained, which may contribute to recrystallization through the particle stimulated nucleation (PSN) mechanism [12,22]. Amounts of sub-micron particles segregated along the grain boundaries. According to research on the Mg-RE alloy [19,23], the fine particles are determined to be β-Mg5RE phases (FCC, a = 2.23 nm) [24], and the present XRD result agrees with these studies (Figure 2d). The β particles dynamically precipitated at grain boundaries exert a Zener pinning effect to impede grain growth during the hot extrusion process. Additionally, dense thin and short lamellar-shaped γ’ phases were observed in the un-DRXed grains, which were first formed during preheating at 400 °C and transformed into 14H LPSO phases with higher heating temperature and longer heating time [25,26].

**Figure 3:**
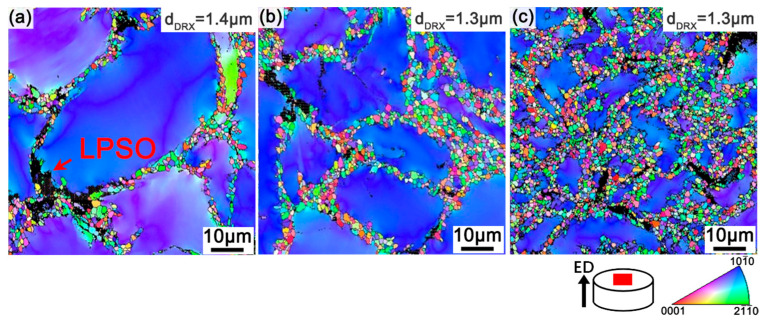
IPF maps of the as-extruded alloys: (**a**) R5, (**b**) R7.5, and (**c**) R10.

The EBSD inverse pole figure (IPF) maps of R5, R7.5, and R10 are shown in Figure 3. The fine DRXed grains exhibit relatively random orientations and the coarse un-DRXed grains have a certain orientation, i.e., //ED. The volume fraction of the DRXed grains is 30%, 43%, and 75% for samples R5, R7.5, and R10, respectively. The DRXed grains were distributed around the un-DRXed grains with a priority, indicating that DRX take place at the original grain boundaries via discontinuous dynamic recrystallization (DDRX) [27]. With extrusion ratio increasing, the higher strain rate drives the DRX process, extending to the un-DRXed grains’ interior via continuous dynamic recrystallization (CDRX) [10]. Additionally, in the IPF maps, the LPSO phases are represented by the black areas, which are not indexed, and some DRXed grains are formed around the LPSO phases. The LPSO phases with a high Young’s modulus bring about the formation of dislocation pile-ups at the interface between the LPSO phases and the soft α-Mg matrix, and then enhance the DRX process by means of the PSN mechanism [28]. The average size of the DRXed grains is 1.4 μm, 1.3 μm, and 1.3 μm, respectively, for samples R5, R7.5, and R10. The DRXed grain sizes of the R7.5 and R10 samples are virtually identical, under the effect of dynamically precipitated β particles. With extrusion ratio increasing, extensive dynamic precipitates form along DRXed grain boundaries, leading to an obvious pinning effect and suppressing the growth of the grains [29].

### 3.3. Tensile Properties

**Figure 4:**
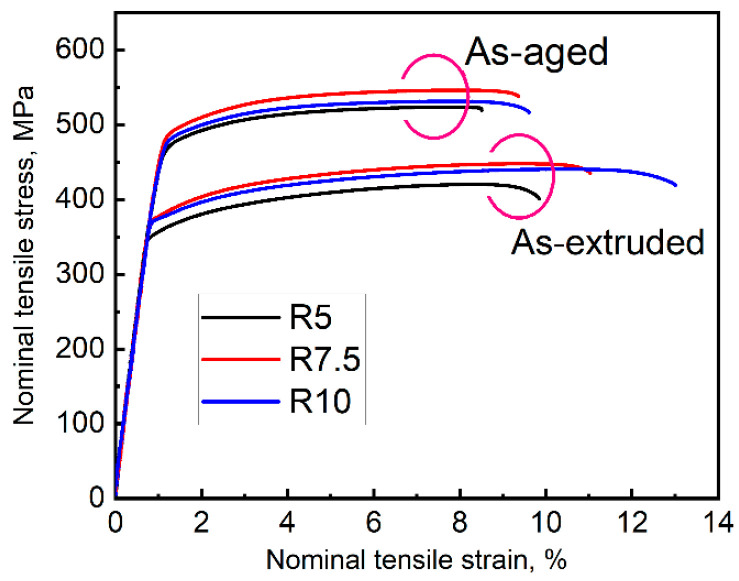
Tensile stress–strain curves of as-extruded and as-aged alloys.

Figure 4 shows the tensile stress–strain curves of the samples. The tensile yield strength (TYS), ultimate tensile strength (UTS), and elongation to failure (EL) are summarized in Table 1. For the as-extruded samples, the R7.5 and R10 samples exhibit higher strength, and the R10 sample shows an excellent EL. Upon ageing, the samples show substantial improvement in strength, and the TYS of R5, R7.5, and R10 samples increases to 465 MPa, 487 MPa, and 477 MPa, respectively, while the EL deteriorates.

Both the R7.5 and R10 samples show good strength values. This is due to the comprehensive effect of the refined grain size, increased DRX fraction, extensive β phases, and uniformly distributed LPSO phases. Firstly, according to the grain refinement mechanism, the fine average DRXed grain size and increased DRX fraction of the R7.5 and R10 samples should contribute to the enhanced strength [30]. Secondly, the high strain rate with increased extrusion ratio accelerates the nucleation and growth of the precipitates [29]. The extensive β particles pinned at the grain boundary inhibit grain boundary slipping and dislocation motion, resulting in dislocation pile-up and causing the improvement of the strength [31,32]. Thirdly, the block-shaped phases are broken under a higher extrusion ratio and distributed evenly over the DRXed regions. The broken phases as a reinforcement significantly strengthen the alloy via short fiber strengthening [33]. The stable coherent interface of the LPSO phases and the α-Mg matrix is also conducive to load transfer and increasing ductility [34]. Thus, the high ductility of the R10 samples is primarily attributed to the uniformly distributed LPSO phases, as well as the increased DRX fraction, and the R10 sample exhibits a superior strength–ductility balance. After ageing, the age-precipitated β’ phases precipitate on the prismatic plane of the α-Mg matrix and hinder the motion of the basal slip, which can significantly strengthen the as-aged alloy [35,36]. However, the micro-cracks prefer to nucleate and propagate at the interface between the β’ precipitates and the α-Mg matrix, and then decrease the ductility [37].

### 3.4. Strain Evolution and Fracture Characterizations

**Figure 5:**
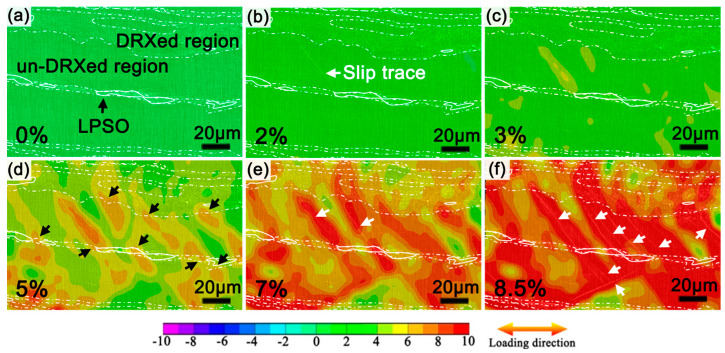
DIC analysis of R7.5 alloy at different macro strains: (**a**) 0%, (**b**) 2%, (**c**) 3%, (**d**) 5%, (**e**) 7%, and (**f**) 8.5%.

To investigate the deformation behavior of the as-extruded alloys, the local strain evolution during plastic deformation and the tensile fracture characteristic of the tensile-tested samples were observed and analyzed. As shown in Figure 5, the local ε_xx_ strain distribution maps of the tensile test sample in the tensile test were obtained via DIC analysis, and the samples were extruded at a ratio of 7.5. The dashed line and solid line represent the interface of the un-DRXed and DRXed regions, and the outline of the LPSO phases, respectively. At a strain of 0–2% (Figure 5a,b), the un-DRXed regions exhibit slight strain localization. At a strain of 2–5% (Figure 5b–d), the local strain rapidly extends in the un-DRXed regions and transfers into the adjacent DRXed regions. The different Young’s modulus and hardness between the LPSO phases and the α-Mg matrix locally cause strain concentrations near the ends of the LPSO phases (marked by black arrows in Figure 5d). With increasing strain (7–8.5%), the local strain transfers across the DRXed regions and LPSO phases and expands outward. Combining the microstructure of the R7.5 sample (Figure 3b), DRXed grains with relatively random orientations are beneficial for releasing localized strain and lead to uniform plasticity. The un-DRXed regions can accommodate a higher strain due to the strong basal texture of (0001)//ED. Some slip traces are observed around the LPSO phases in the un-DRXed regions, marked by white arrows (Figure 5 b,e,f). The LPSO phases can effectively inhibit the basal slip and result in stress concentration between the LPSO phases and the un-DRXed regions, and then inspire the formation of slip traces and strengthen the alloys [38].

**Figure 6:**
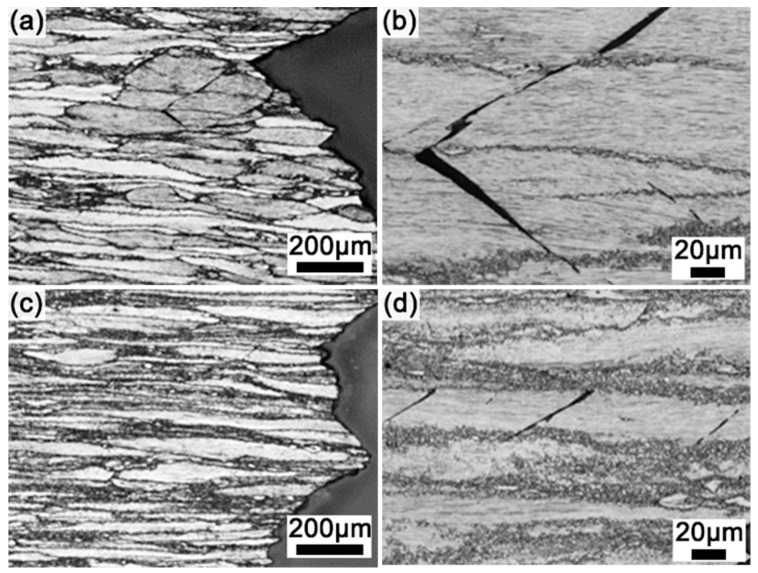
Fracture characteristics of the fractured samples: (**a**,**b**) R5 and (**c**,**d**) R10.

**Figure 7:**
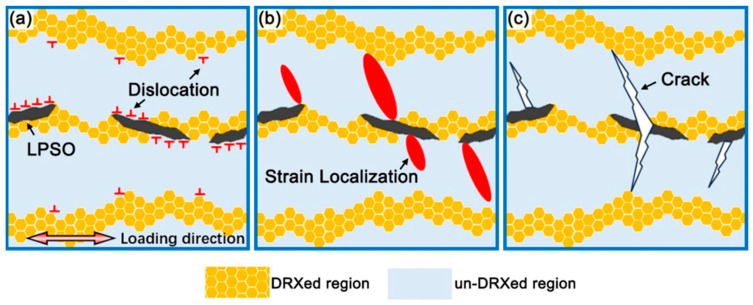
Schematic illustration of the deformation process of as-extruded samples during tensile test.

Figure 6 shows the fracture characteristics of the fractured samples. It is obvious that the micro-cracks in the R5 sample cross the un-DRXed and DRXed regions, while the micro-cracks are limited in the un-DRXed regions in the R10 sample. The length of micro-cracks is ~80 μm and 40 μm for the R5 and R10 samples, respectively. The schematic diagram of the deformation process of the as-extruded alloys is shown in Figure 7. The above discussion shows that due to the mismatch of Young’s modulus and deformation incompatibility, the strain is mainly located at the interface of different regions (un-DRXed regions, DRXed regions, and LPSO phases) (Figure 7a). The DRXed regions and the LPSO phases release localized strain by means of grain rotation and kinking deformation, respectively [28,39]. Strain localization induces crack initiation and propagation in the un-DRXed regions (Figure 7b,c) [40]. The higher DRXed volume fraction and decreased thickness of the un-DRXed grains of the R10 sample effectively suppress crack propagation, which contributes to the excellent ductility of the R10 sample. Therefore, further enhancement of the strength and ductility in Mg-RE alloys with LPSO phases can be accomplished by modifying the bimodal microstructure and distributions of their components.

## 4. Conclusions

In this work, Mg-Gd-Y-Zn-Zr alloys were hot-extruded with various extrusion ratio, and further treated with ageing treatment. The mechanism of a bimodal microstructure and LPSO phases on the mechanical properties of the Mg-RE-Zn alloys was systematically studied. The main conclusions are as follows:

1. Regardless of the extrusion conditions, the bimodal microstructure comprises elongated un-DRXed grains, fine DRXed grains, β particles, nano-sized γ’ phases, and LPSO phases in all as-extruded alloys. A larger extrusion ratio induces a higher strain rate and finer LPSO phases, and hence leads to a higher volume fraction of dynamic recrystallization. Meanwhile, because of the pinning effect of extensive β particles, the size of the DRXed grains changes slightly. Both the DDRX behavior and the CDRX behavior contribute to the dynamic recrystallization process.
2. The sample extruded with a ratio of 10 exhibits an excellent strength–ductility synergy with a TYS of 374 MPa, a UTS of 440 MPa, and an EL of 13.0%, which is mainly attributed to its fine DRXed grains, extensive β particles, and elongated block-shaped LPSO phases. After ageing treatment, the strength further increases, due to the precipitation strengthening effect induced by β’ phases.
3. During the deformation process, the un-DRXed regions firstly show strain localization, and DRXed grains with relatively random orientations can effectively release localized strain and prevent the expansion of micro-cracks, thus promoting uniform plastic deformation and improving the ductility of the alloy. Due to the mismatch of Young’s modulus and deformation incompatibility, the micro-cracks primarily nucleate at the interface of the un-DRXed regions, DRXed regions, and LPSO phases, and then propagate in the un-DRXed regions.

## Figures and Tables

**Table 1:** Mechanical properties of the studied alloys at room temperature.

Sample	As-Extruded			As-Aged		
	TYS (MPa)	UTS (MPa)	EL (%)	TYS (MPa)	UTS (MPa)	EL (%)
R5	356	420	9.8	465	524	8.6
R7.5	379	448	11.0	487	546	9.4
R10	374	440	13.0	477	532	9.7

## Data Availability

The original contributions presented in the study are included in the article, further inquiries can be directed to the corresponding author.
